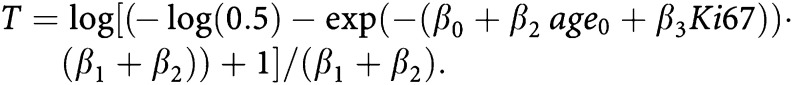# The Ki67 index a prognostic marker in medullary thyroid carcinoma

**DOI:** 10.1038/sj.bjc.6601453

**Published:** 2003-11-25

**Authors:** L E Tisell, A Oden, A Muth, G Altiparmak, J Mõlne, H Ahlman, O Nilsson

**Affiliations:** 1 The Lundberg Laboratory for Cancer Research, Department of Surgery, Göteborg University Hospital, Göteborg University, Gothenburg, Sweden; 2 Department of Pathology, Göteborg University Hospital, Göteborg University, Gothenburg, Sweden; 3 Department of Mathematics and Statistics, Göteborg University Hospital, Göteborg University, Gothenburg, Sweden

**Keywords:** MTC, proliferation index, survival

## Abstract

Our objective was to examine the usefulness of the Ki67 proliferation index as a prognostic marker in patients with medullary thyroid carcinoma (MTC). It is difficult to predict the prognosis of MTC by using conventional prognostic factors. Immunocytochemical analysis of tumour proliferation has been used as a prognostic tool in some tumours, but only rarely in MTC. In all, 71 tumours from 36 patients were investigated, by using a semiautomatic image analysis programme. On average 10 000 nuclear profiles were counted per tumour, and the percentage of tumour cells expressing the proliferation marker, Ki67, was calculated. Primary tumours that had metastasised had higher Ki67 indices than primary tumours that had not metastasised. Recurrent lymph node metastasis had higher Ki67 indices than the primary tumours. By using a Poisson model, it was possible to estimate the median survival time for *individual* patients if the Ki67 index for the primary tumour and the age at surgery were known. The higher the Ki67 index and the age at operation were, the shorter was the survival. Estimating the median survival of *individual* patients will be of help for planning the patients' life and postoperative follow-up and treatment.

Medullary thyroid carcinoma (MTC) is derived from the calcitonin-producing C cells of the thyroid. Determination of serum calcitonin concentrations is used to diagnose and follow patients with MTC (Wells *et al*, 1978; Tisell *et al*, 1996). In the TNM classification of thyroid tumours, papillary carcinoma with lymph node metastasis in young patients is classified as a stage I disease. A similar extent of MTC has a worse impact on survival and is classified as a stage III disease (The Committee on TNM classification UICC, 1992). Medullary thyroid carcinoma patients with lymph node metastasis often have persistent disease after the operation. In one-third of such patients, meticulous neck dissection led to normalisation of the serum calcitonin concentrations (Tisell *et al*, 1986; Moley *et al*, 1993). The prognosis of MTC patients is difficult to predict using conventional prognostic factors, for example, age, sex, stage, histopathological growth pattern, ploidy or biochemical tumour markers (Tisell *et al*, 1996). Half of the MTC tumours can be localised with octreotide scintigraphy due to their expression of somatostatin receptors. The visualised tumours grew more rapidly and aggressively than nonvisualised lesions, suggesting that octreotide scintigraphy may be used as a prognostic tool (Tisell *et al*, 1997). Immunocytochemical analysis of tumour proliferation has become an important tool for the assessment of tumour aggressiveness and prognosis (Tubiana and Courdi, 1989). The aim of the present study was to evaluate tumour proliferation, measured by Ki67, as a prognostic marker for MTC. For this, Ki67 indices of primary tumours were related to the clinical course of the MTC patients. The possible presence of mutation of the p53 gene was also investigated.

## MATERIAL AND METHODS

### Patients

Paraffin blocks with tumour tissue from 36 patients, 20 females and 16 males were retrieved. Each patient had at least one operation for MTC at the Sahlgrenska University Hospital. In all, 23 patients had sporadic MTC, 12 had MEN 2A and one a MEN 2B syndrome. The mean age (s.d.) at operation was 45 (15) years (median 44years). The tumours were classified according to the TNM classification (The Committee on TNM classification UICC, 1992).Tumour stage at initial operation was: stage I, two patients; stage II, six patients; stage III, 22 patients; and stage IV, six patients. The follow-up ended in March 2000. At diagnosis, six patients had distant metastasis. During follow-up 11 additional patients got distant metastasis. In all, 13 of the patients with distant metastasis died of MTC at a mean (s.d.) of 8 (7) years (median 7 years) after diagnosis, two patients died of unrelated causes after 17 years. A total of 21 patients were alive at follow-up at a mean of 20 (9) years (median 18 years) after the operation. All survivors were asymptomatic, but four had distant metastasis. The postoperative observation time for the total series was 15 (10) years (median 15 years). Individual clinical features of the 36 patients can be found in Table 1

**Table 1:** Clinical features of the 36 MTC patients

**No.**	**Sex**	**MTC type**	**Ki67% primary tumour**	**Tumour diameter (mm)**	**Tumour stage pTNM**	**Age at operation (years)**	**Postoperative survival (years)**	**Status at follow-up**
1	F	H^*^	0.01	5	III	24.89	14.06	Alive
2	M	S	0.03	18	III	59.16	17.26	Died O.C.†
3	F	H	0.05	60	II	35.14	49.11	Alive
4	M	S	0.09	27	II	43.75	26.13	Alive
5	M	S	0.19	50	III	49.90	17.25	Died O.C.†
6	F	S	0.20	10	III	41.41	26.91	Alive
7	F	S	0.23	45	IV	29.30	19.19	Died MTC
8	M	H	0.24	55	II	31.95	14.23	Alive
9	M	H	0.24	4	I	18.73	22.69	Alive
10	F	S	0.26	5	I	48.61	18.18	Alive
11	F	S	0.26	22	III	74.19	8.61	Died MTC
12	F	S	0.27	13	III	60.84	29.61	Alive
13	F	S	0.30	20	II	55.29	18.60	Alive
14	F	H	0.31	5	III	31.55	16.08	Alive
15	F	S	0.32	40	III	38.65	24.40	Alive
16	M	H	0.33	60	III	41.84	6.49	Died MTC
17	M	H	0.34	20	IV	59.32	2.65	Died MTC
18	M	H	0.36	47	III	30.06	22.60	Died MTC
19	F	H	0.46	13	III	42.38	12.77	Alive
20	F	S	0.46	40	IV	30.84	5.07	Died MTC
21	F	H	0.49	30	II	22.19	10.61	Alive
22	F	H	0.56	25	III	38.13	13.06	Alive
23	F	S	0.59	42	III	50.64	9.54	Died MTC
24	F	S	0.62	45	II	66.43	6.95	Died MTC
25	M	S	0.63	45	III	38.41	20.07	Alive
26	F	H	0.73	15	III	65.38	10.11	Alive
27	F	H	0.75	4	III	18.58	16.30	Alive
28	M	S	0.77	40	III	47.56	10.05	Died MTC
29	F	S	0.85	25	III	39.81	19.71	Alive
30	M	S	0.88	12	III	63.84	12.38	Alive
31	M	S	1.12	50	III	43.45	25.32	Alive
32	F	S	1.27	47	III	62.49	16.6	Alive
33	M	S	1.84	70	III	59.16	7.65	Died MTC
34	M	S	2.58	Missing	IV	56.78	1.79	Died MTC
35	M	S	3.51	37	IV	64.27	1.09	Died MTC
36	M	S	4.5	50	IV	47.72	0.64	Died MTC

H=hereditary; MTC, S=sporadic MTC; died O.C.

† =died of other causes than MTC; pTNM=postsurgical histopathological classsification; T=tumour; N=regional lymph node metastasis; m=distant metastasis.

.

In all, 71 tumour specimens were investigated, 36 primary tumours, 21 primary lymph node metastasis and 14 recurrent lymph node metastasis. The mean (s.d.) (median) of the Ki67 indices for all the 71 tumours was 1.71% (3.3) (median 0.61%), the Ki67 indices of the primary tumours, primary metastasis and recurrent metastasis were 0.74% (0.96) (median 0.41%), 1.35% (1.49) (median 0.85%) and 4.73% (6.39) (median 1.65%), respectively. In 12 cases, tumour tissue was available from primary tumours, primary metastasis and also from recurrent metastasis. This subset of patients was tested for within-subject differences between the Ki67 indices of the three kinds of tumours. In another analysis, the Ki67 values of the 28 primary tumours that had metastasised were compared to those of the eight tumours that had not metastasised.

### Therapy

Since 1972, the operation involved a total thyroidectomy and a meticulous central lymph node dissection. Lateral lymph node dissections, on one or both sides were carried out in cases with palpable primary tumours or palpable lymph nodes in the central or lateral neck. Patients cared for at our hospital, after less radical operations performed before 1972, or after incomplete surgery elsewhere, had completion thyroidectomies with central lymph node dissections. In case of tumour recurrence repeat neck operations were performed. After the operations, the patients were followed annually at our outpatient clinic. Patients with terminal disease were followed more often. The close surveillance of the patients guaranteed that the causes of death could be verified in all cases.

### Immunocytochemistry

Paraffin blocks of tumour tissue were retrieved from the Department of Pathology, Sahlgrenska University Hospital. The tissues had been routinely fixed in neutral-buffered formalin for 1–2 days and embedded in paraffin wax. Sections (3–4 *μ*m) from primary tumours and lymph node metastasis were placed on positively charged glass slides, deparaffinised and rehydrated. Antigens were retrieved by microwave treatment in citrate buffer (10 mM citrate, pH 6.0) twice for 5 min. In order to assure even staining of tissue sections, the incubation of sections was performed in a Techmate 500 immunostainer under identical conditions. Incubation with primary antibodies was carried out for 25 min, followed by biotinylated goat anti-mouse antibodies (LSAB, Dako), streptavidin–HRP and diaminobenzidine. After counter staining in Mayer's haematoxylin, sections were dehydrated and mounted. All antisera were diluted in antibody diluent (Tris-buffer pH 7.2, containing 15 mM sodium azide and protein; Dako). The following primary antibodies were used: mouse anti-Ki67 monoclonal antibody (clone MIB-1; Immunotech) and mouse anti-p53 monoclonal antibodies (clone PAb1801 and DO-7; Dako). As negative controls served sections incubated identically, except for the primary antibody that was replaced by normal mouse IgG. As positive controls served sections of normal tissues (lymph node for Ki67) or tumour tissues (colorectal cancer for p53).

### Determination of proliferation indices

In order to estimate the growth rate of tumours, the percentage of tumour cells expressing the proliferation marker Ki67 was measured. A proliferation index was calculated for each tumour lesion by counting the total number of tumour cell nuclear profiles and the number of Ki67-positive nuclear profiles in randomly and systematically selected fields. The first field in each tumour lesion was selected randomly, and the following fields were sampled systematically using a mesh. On average 10 000 nuclear profiles were counted per tumour lesion. Counting was performed using a semiautomatic image analysis programme (IBAS).

### Statistics

The Mann–Whitney test was used to compare the Ki67 values of the primary tumours that had not metastasised (stages I and II) with those that had metastasised (stages III and IV). The Friedman test followed by Dunn's multiple comparison post-test was used to investigate within-subject differences between Ki67 values of primary tumours and primary and recurrent metastasis of the 12 patients who had tumour tissue available from all the three kinds of tumour lesions.

#### Survival analysis

For the survival analysis, the following variables were noted for each patient: Ki67 index of the *primary* tumour, age at operation, survival time after the operation, death of MTC or not (Table 1). A Poisson model (Breslow and Day, 1987) was applied to estimate the hazard function of death from MTC. The hazard function was of the form exp (*β*_0_+*β*_1_ × time since operation+*β*_2_ × current age+*β*_3_ × Ki67) Also a model including interaction between time and Ki67 was studied. For the derivation of the cause-specific median survival time see the addendum. If the age at operation and the Ki67 index are known, the formula of the last line of the addendum can be used to calculate the median survivals for individual patients.

In a Poisson regression, the variables tumour size, sex, type of tumour and tumour stage were included one at a time together with the variables Ki67 and age to find out if any of the four first mentioned variables had any significant importance for death of MTC. For these calculations, tumour size was represented by the largest tumour diameter, type of tumour was sporadic or hereditary tumours, stage was stages I and II or stage III and IV. The variables for each patient can be found in Table 1. The two patients who died of unrelated causes were censored at the date of death and the patients alive at the end of the study were censored at that time.

## RESULTS

The subset of 12 patients who had tumour tissue available from primary tumours, primary metastasis and recurrent metastasis could be used to test for within-subject differences between the three kinds of tumour lesions. In this subset of patients, the Ki67 values of the primary tumours, the primary metastasis and the recurrent metastasis were 0.80% (0.74) (median 0.53%), 1.56% (1.72) (median 1.11%) and 4.62% (6.40) (median 1.65%), respectively. The higher value for the recurrent metastasis *vs* the primary tumours was significant (*P*<0.01).

Primary tumours that had metastasised, stages III and IV, had higher Ki67 indices, 0.87% (1.05) (0.51%) than primary tumours without metastasis, stages I and II, 0.29% (0.19) (0.25%) (*P*<0.05).

### Survival analysis

The *β*-coefficients determining the hazard function and the median postoperative survival time are found in Table 2

**Table 2:** *β*-coefficients for calculating the hazard function and the estimated median survival time

**Variable**	***β*-coefficients**	**s.e.**	**Two-tailed *P*-value**
Constant	−5.9290	1.3405	
Time since operation (years)	−0.0199	0.0436	*P*>0.30
Current age (years)	0.0285	0.0224	*P*=0.22
Ki67%	1.1347	0.2437	*P*<0.001

. The estimated median cause-specific survival times, at three ages, for five different Ki67 values, are presented in Table 3

**Table 3:** The estimated postoperative median survival for five Ki67 values at three ages

**Ki67 index of primary MTC tumours**	**Age at operation (years)**	**Cause-specific, median survival (years)**
0.6	35	40.6
0.7	35	36.8
1.0	35	27.4
2.0	35	9.5
4.5	35	0.6
		
0.6	45	31.8
0.7	45	28.2
1.0	45	21.2
2.0	45	7.2
4.5	45	0.4
0.6	55	24.7
0.7	55	22.2
1.0	55	16.3
2.0	55	5.5
4.5	55	0.3

Note that the higher the Ki67 index of the primary tumour and age at operation were, the shorter was the estimated postoperative survival.

. This table shows that the estimated median survival time will be reduced with higher ages at surgery and with higher Ki67 indices. Median survival times for other values of age and Ki67 can be calculated by using the formula on the last line of the addendum.

Figure 1

**Figure 1:**
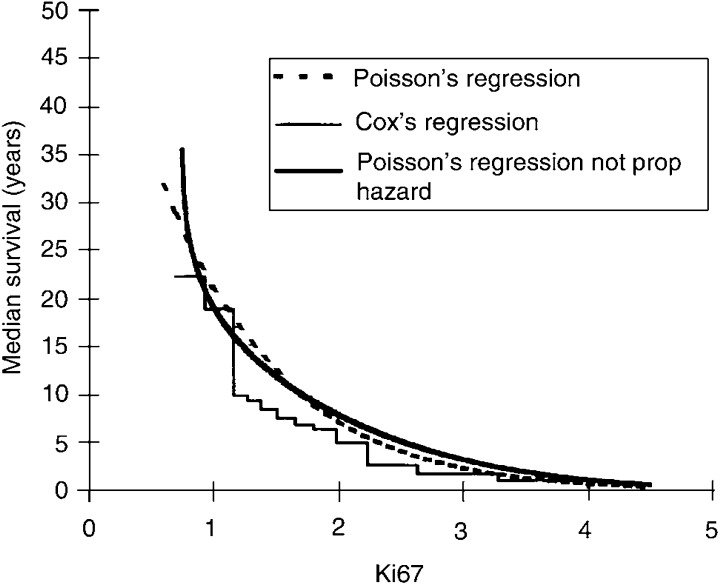
The figure shows as an example, the calculated curves of median survival where the age at operation is 45 years. The three curves show similar relations between the median survival and the Ki67 index. The curve of the Cox regression is a step function and from the figure it is apparent that this is a weakness when it is applied to individual patients. The Poisson regression with nonproportional hazard, which includes interaction between Ki67 index and time, does not differ significantly from the simpler Poisson model. By use of the Poisson regression result, the median survival of individual patients can be calculated. The variables entered in the three regression curves were original data from the studied population.

shows an example of three calculated curves of median survival, where the age at operation is 45 years. In contrast to the curves of the Poisson regressions is the curve of the Cox regression a step function, which is a weakness when applied to individual patients. The variables entered in the three regression curves were original data from the studied population.

When including the variables sex, tumour size, tumour type and tumour stage, one at a time together with Ki67 and age, in a Poisson regression none of the four first mentioned variables had any significant importance for death of tumour. Closest to significance was size with *P*=0.1710. The calculation of median survival depending on Ki67 was not affected by the new variables.

### p53 labelling

Immunostaining with two different antibodies against p53 failed to label any of the investigated tumours, while control tissues (colorectal carcinoma) were positive. This is in accordance with previous studies showing the absence of p53 mutations in MTC (Yana *et al*, 1992; Herfarth *et al*, 1997).

## DISCUSSION

Several studies have investigated the usefulness of proliferation markers as prognostic tools in human neoplasm, for example, soft-tissue tumours, prostate, ovarian and lung carcinoma (Raymond *et al*, 1988; Pence *et al*, 1993; Drobnjak *et al*, 1994; Kerns *et al*, 1994). Immunocytochemical detection of the nuclear proliferation antigen Ki67 with MIB-1 monoclonal antibody can be used to study proliferation in paraffin-embedded tumour material. This method has been used also in endocrine tumours. For thyroid tumours, it has been shown that anaplastic tumours have higher Ki67 indices than well-differentiated thyroid tumours (Carr *et al*, 1993), and that Hürthle cell carcinomas of the thyroid have higher Ki67 indices than benign Hürthle cell adenomas (Erickson *et al*, 2000). There are few studies about Ki67 indices in MTC, and these studies include few patients (Rigaud and Bogomoletz, 1991; Carr *et al*, 1993; Wang *et al*, 1996). In the present long-term follow-up study, we examined MTC tumour specimens from 36 patients to find out if the level of Ki67 expression was related to the level of malignancy. According to the TNM system, MTC tumours without metastasis are classified as stages I and II disease, while tumours with metastasis are classified as stages III and IV disease. Significantly higher Ki67 indices were found in primary tumours that had metastasised than in primary tumours without metastasis. It was also found that recurrent metastasis had higher Ki67 indices than primary tumours, which is in agreement with the observation that in papillary thyroid cancer the proportion of proliferating cells increased with each recurrence (Ozaki *et al*, 1999).

According to DeLellis (1995), the Ki67 index cannot be used to judge the malignancy of individual endocrine tumours, but this marker may be helpful in identifying subsets of tumours that will follow an aggressive course. To explore whether the Ki67 index could predict the cause-specific postoperative survival in individual patients, we used a Poisson model to analyse the data (Breslow and Day, 1987) and a formula was derived that can be used to estimate the cause-specific median survival time for individual patients, if the Ki67 index and age at surgery are known. Estimated individual survival times were found to be reduced with higher Ki67 indices and higher age at surgery. With the Poisson model, the estimated median survival can be obtained for every age and for every value of the Ki67 index. The median survival can also be calculated with the help of a Cox model, but compared to the Poisson model it is less appropriate since the resulting function of the Cox model is a step function, which cannot be used to obtain the median survival for every value of the Ki67 index (cf Figure 1). When including the variables tumour size, sex, type of tumour (sporadic or hereditary) and tumour stage, one at a time together with Ki67 and age, in a Poisson regression none of the four first mentioned variables had any significant importance for death of MTC. The calculation of median survival depending on Ki67 was not affected by the new variables. This shows that the Ki67 index is a very suitable prognostic marker for MTC.

Estimating the median survival of *individual* patients will be of help for planning the patients' life and postoperative follow-up and treatment.

## ADDENDUM

**Derivation of the median survival time *T***.

Let *h(t)* be the hazard function of death.

Then 
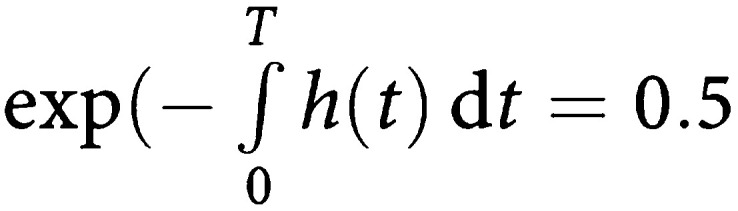


Thus 
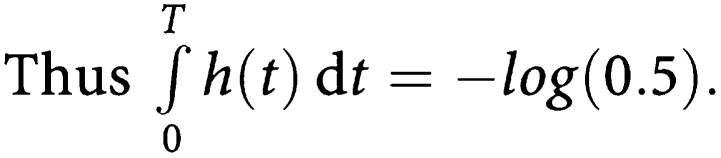


Let age_0_ be the age at operation. Then the current age *t* years after operation is age_0_+*t*.

Hence, the above equality can be written:


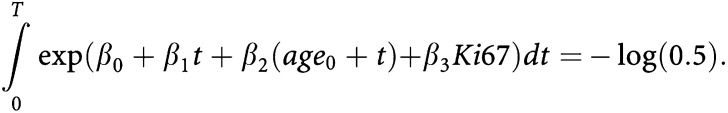


Then we have 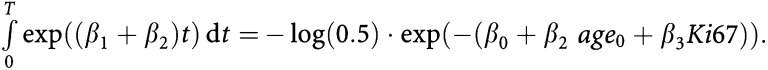


Then the integral can be calculated and thus


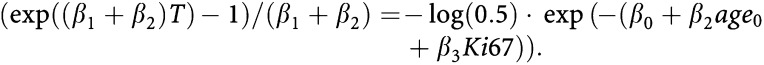


Finally,